# Supplementary material for: Curriculum learning for ab initio deep learned refractive optics
Source: Nat Commun. 2024 Aug 3;15:6572. doi: 10.1038/s41467-024-50835-7 (PMC11297943; doi:10.1038/s41467-024-50835-7)
Supplement: Supplementary file 1 — Supplymentary Information [file 41467_2024_50835_MOESM1_ESM.pdf]

Supplemental Material for: Curriculum Learning for ab initio Deep Learned Refractive Optics

Xinge Yang[1], Qiang Fu[1], and Wolfgang Heidrich[1,*]

[1]King Abdullah University of Science and Technology (KAUST), Saudi Arabia
[*]Corresponding author: wolfgang.heidrich@kaust.edu.sa

Supplementary Note 1 Technical Details of Differentiable Ray-tracing

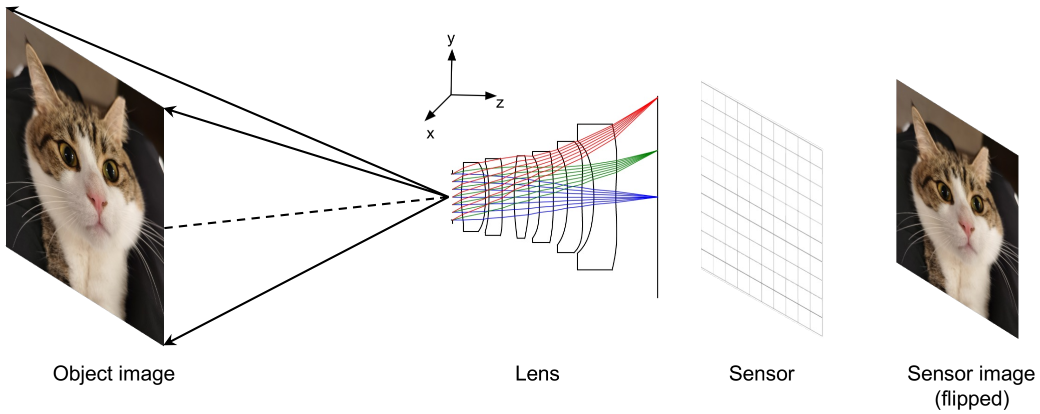

Supplementary Figure 1. Our ray-tracing imaging system. The imaging system consists of a 2D RGB image, an optical lens, and an image sensor. Optical rays start from sensor pixels and pass through the lens. When optical rays hit the object image, we do bilinear interpolation to calculate the color of each ray and simulate the sensor image.

Differentiable ray tracing[1,2] provides a perspective on optical design by directly optimizing the final image quality. As shown in Fig. S1, we use a ray tracing technique to simulate the sensor image. Optical rays start from sensor pixels and propagate through the lens via a sequence of intersection and refraction with the lens surfaces. When rays intersect with the 2D object image, we calculate the color of each ray by interpolating the ray position. Then we do Monte-Carlo numerical integration to simulate the sensor image. The ray tracing rendering process is designed to be continuous and differentiable, so we can back-propagate the image error to learn the lens parameters like curvature and position.

An optical ray can be expressed as

$$ray = (\mathbf{o}, \mathbf{d}, \lambda), \quad s.t. \quad ||\mathbf{d}||_2^2 = 1, \tag{1}$$

where position $\mathbf{o}$ and direction $\mathbf{d}$ are 3D vectors and $\mathbf{d}$ is normalized to have unit length. The wavelength λ is a float number that is used to take into account the dispersion of the optical lens. We discrete the full spectrum into three wavelengths (486 nm, 587 nm, and 656 nm) in our experiments. At the beginning of the differentiable ray tracing process, we sample rays from sensor pixels. Please refer to Supplementary Note 3 for detailed experiment settings.

The ray propagation within the lens can be decomposed into a series of intersections and refractions with each optical surface. Note that differentiable ray tracing can be used for any type of continuous surface, but in our experiments, we mainly consider aspheric surfaces

$$z(r) = \frac{r^2}{R\left(1+\sqrt{1-(1+\kappa)\frac{r^2}{R^2}}\right)} + \alpha_2 r^2 + \alpha_4 r^4 + \cdots, \tag{2}$$

where the optic axis is presumed to lie in the z axis, and $z(r)$ is the *sag* — the z-component of the displacement of the surface from the vertex at distance $r = \sqrt{x^2+y^2}$ from the axis. R is the radius of curvature (*roc*), and we often use its reciprocal curvature c in the practice. κ is conic, and the polynomial coefficients α_i describe the deviation of the surface from the axially symmetric quadric surface. In subsequent calculations, for the sake of generality, we will use the shorthand notation

$$\mathcal{S}(x, y, z) = 0. \tag{3}$$

The intersection position of a ray and an optical surface can be solved by

$$\mathbf{o}' = \mathcal{I}(\mathbf{o}, \mathbf{d}, \mathcal{S}) \iff \begin{cases} \mathbf{o}' = \mathbf{o} + \mathbf{d}\cdot t \\ \mathcal{S}(o'_x, o'_y, o'_z) = 0 \end{cases}, \tag{4}$$

where $\mathbf{o}$, $\mathbf{d}$ represent the original position and direction of the incident ray, and $\mathbf{o}'$ represents the intersection position, as shown in Fig S2a. Especially, for a spherical surface where k and α_i equal to 0 in Eq. (2), an explicit solution exists for Eq. (4). But for an aspheric surface where an explicit solution does not exist, we have to iteratively find the root by Newton's method. The pseudo-code is shown in Algorithm 1.

Algorithm 1 Newton's method to solve intersection equation

```
t ← t_0
while N < N_0 do
    t ← val(t)                                  ▷ Validation check
    o ← o + d · t                               ▷ Update point
    s ← S(o_x, o_y, o_z)                        ▷ Surface residual
    dsdt ← S'(o_x, o_y, o_z) · (ȯ_x, ȯ_z, ȯ_z)   ▷ Surface derivative using chain rule
    δt ← min(s/dsdt, δt_0)                      ▷ Newton step bound
    t ← t − δt                                  ▷ Newton iteration
end while
o' ← o + d · t
```

The implementation of Newton's method Algorithm 1 follows[2], while a validation check is added during Newton's iteration to improve the stability of the calculation. Due to the spherical part of aspheric surfaces, the (x, y) coordinates have a valid range. If the updated (x, y) exceeds this range, a NaN error will be generated, and both the forward and backward passes will be crashed. Therefore, each time before updating $\mathbf{o}$, we do a validation check and then update only those points that are valid. In the experiment, we use a small δt_0 (usually 3) to constrain the step size of Newton' s method, because the shape of the aspheric surface usually changes very drastically. To reduce memory consumption during the calculation, the first $N_0 - 1$ iterations are executed without gradient tracking. We only track the gradient information at the last iteration, which is the same as[2]. Even though the gradient calculated with this method is biased, experimental results show that it can lead to final convergence and success.

When passing through the boundary of two different mediums, the optical ray will refract and change direction, as shown in Fig. S2a. This refraction process can be formulated by Snell's law

$$\mathbf{d}' = \mathcal{R}(\mathbf{o}', \mathbf{d}, \mathcal{S}) \iff \begin{cases} \mathbf{n} = \nabla\mathcal{S} \\ \mathbf{n}\times\mathbf{d}' = \mu(\lambda)\mathbf{n}\times\mathbf{d}, \end{cases}, \tag{5}$$

where $\mathbf{n}$ is the surface normal vector, $\mathbf{d}'$ represents the refractive direction. $\mu(\lambda)$ is the refractive index related to medium material and wavelength. The refractive index can be calculated by empirical dispersion formulas like Sellmeier equation[3,4]

$$\mu^2(\lambda) = 1 + \frac{B_1\lambda^2}{\lambda^2 - C_1} + \frac{B_2\lambda^2}{\lambda^2 - C_2} + \frac{B_3\lambda^2}{\lambda^2 - C_3}, \tag{6}$$

where $B_1, B_2, B_3, C_1, C_2, C_3$ are Sellmeier coefficients of the material.

Solving Eq. (5) yields an explicit expression of $\mathbf{d}'$

$$\mathbf{d}' = \sqrt{1 - \mu(\lambda)^2\left[1 - (\mathbf{n}\cdot\mathbf{d})^2\right]}\mathbf{n} + \mu(\lambda)[\mathbf{d} - (\mathbf{n}\cdot\mathbf{d})\mathbf{n}]. \tag{7}$$

It is worth noting if $\mathbf{d}$ and $\mathbf{n}$ are normalized then $\mathbf{d}'$ is also normalized. We encourage readers to refer to[5,6] for derivation of Eq. (7).

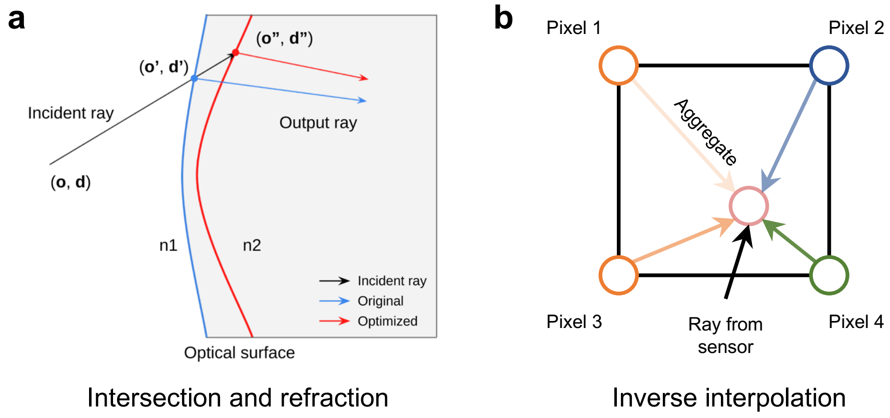

Supplementary Figure 2. Three calculation steps in differentiable ray tracing. The differentiable ray tracing process consists of a series of intersections and refractions, and finally interpolation on the object image. **a** Blue curve. In forward pass, the ray $(\mathbf{o}, \mathbf{d})$ intersects the lens surface($\mathbf{o}'$) and is refracted($\mathbf{d}'$) as it passes through the surface. Red curve: In the backward pass, the back-propagated gradient updates the lens surface to control the intersection position($\mathbf{o}''$) and the output ray direction($\mathbf{d}''$). **b** In forward pass, we interpolate the intersection position($\mathbf{o}$) on the object image to obtain the color of each ray. In the backward pass, the image error is back-propagated and the ray is directed to the correct pixel ($\mathbf{o}'$, red arrow).

After passing through the lens, the ray propagates to the object plane. We do a bilinear interpolation on the 2D object image to calculate the color of the ray

$$\mathcal{B}(\mathbf{o}, I) = \begin{bmatrix} 1-\omega_i, & \omega_i \end{bmatrix} \begin{bmatrix} I(0,0), & I(0,1) \\ I(1,0), & I(1,1) \end{bmatrix} \begin{bmatrix} 1-\omega_j \\ \omega_j \end{bmatrix}, \tag{8}$$

where I is the 2D object image, and $\mathbf{o}$ is the intersection position on the object plane, as shown in Fig. S2b. We gather neighboring four pixels of the intersection point and do interpolation to calculate the illuminance of the ray. ω_i, ω_j are the horizontal and vertical distance from the intersection point to pixel $I(0,0)$. Although a 2D image is not an accurate representation of a real 3D scene, it has been shown to be useful for many depth-independent applications[1,7–9]. We simulate the sensor-captured image with Monte-Carlo numerical integration.

$$\widetilde{I}(s,t) = \frac{1}{spp}\sum_{k=1}^{spp} \mathcal{B}(\mathbf{o}_k(s,t)), \tag{9}$$

where $\widetilde{I}$ is the simulated sensor image, (s,t) is the pixel coordinates. The sensor has the same resolution as the object image, and we scale the physical size of the object image so the simulated image can cover the full sensor and align with the object image pixel-by-pixel after a flipping operation. For detailed experimental implementations, please refer to Supplementary Note 3.

In Eq. (9), only valid rays will contribute to the simulation, so the sensor image will be slightly darker than the object image, especially at the corner. However, this vignetting effect does not provide valid gradients in backpropagation, so we only average on valid rays to remove vignetting. Combining Eqs. (4), (5), (8) and (9), we get

$$\widetilde{I} = \mathcal{B}\left(\mathcal{R}_M \mathcal{I}_M \mathcal{R}_{M-1} \mathcal{I}_{M-1} \cdots \mathcal{R}_1 \mathcal{I}_1(\mathbf{o}, \mathbf{d}), I\right), \tag{10}$$

where M is the number of optical surfaces. Eq. (10) suggests that the forward rendering process can be decomposed in a sequence of continuous and differentiable operations, which allows us to back-propagate gradients from image pixels to the lens parameters. In the back-propagation, the ray position $\mathbf{o}$ and direction $\mathbf{d}$ work as intermediate variables to pass the gradient between the different surfaces, as shown in Fig. S2a-b. Gradients from sensor pixels $\widetilde{I}$ with respect to the lens parameters θ, *i.e.* $\frac{\partial \widetilde{I}}{\partial \theta}$, can be computed via

$$\frac{\partial \widetilde{I}}{\partial \theta} = \frac{\partial \widetilde{I}}{\partial \mathbf{o}} \frac{\partial \mathbf{o}}{\partial \theta} + \frac{\partial \widetilde{I}}{\partial \mathbf{d}} \frac{\partial \mathbf{d}}{\partial \theta}. \tag{11}$$

We developed our differentiable ray tracing model based on[2], while a lot of implementation details are optimized for better performance. Till now, the most basic differentiable model can be used to design lenses, and we can directly minimize the pixel-level difference between the object image and the simulated image. The loss function can be then written as

$$\mathcal{L} = \|\widetilde{I} - I\|_2^2. \tag{12}$$

Ideally, the rays from the sensor pixels will converge to the corresponding pixel in the object plane. Thus, in backpropagation, the gradients push the rays toward the correct pixel. The backpropagation can be understood like this: the interpolation gradients guide the rays to move toward the correct pixels, and interpolation and refraction gradients bend the optical surfaces to control ray directions. It is worth noting that, although Eq. (8) requires the ray to fall into the correct square for correct gradients, the low-frequency information in the image can also guide the ray to move towards the correct direction when it falls outside.

Supplementary Note 2 Proposed Methods for Differentiable Ray-tracing

The basic differentiable ray tracing approach proposed in[1,2] cannot address the significant memory consumption problem during the optimization of compound lenses with high-resolution images. Additionally, the pixel-based image loss consistently corrects lens distortion during optimization, which is not always desired, especially when designing lenses with a large FoV. In this section, we introduce several new features to tackle this issue, including adjoint rendering, shape correction, and distortion relaxation.

Supplementary Note 2.1 Adjoint Rendering

Memory consumption has long been a problem for differentiable ray-tracing rendering. While existing methods either compute adjoint derivatives in the forward pass[10,11], recompute the optical path[12], or simply use small sensor resolution and spp[1]. The adjoint derivative calculation method[10,11] can greatly reduce memory consumption, but its implementation is not flexible. In particular, if we change learnable parameters or experimental settings, we have to recalculate the adjoint derivative. Like[12], we propose a so-called "adjoint rendering" approach to solve the memory problem, while being easy to implement and applicable to any different optical system.

See Fig. S3, where adjoint rendering separates the backpropagation process to reduce memory consumption without affecting gradients. Like[12], the core idea is to re-do the differentiable raytracing to compute the lens gradient. In practice, we first simulate the sensor image and feed it to the downstream reconstruction network without recording the gradient information during ray tracing. This step is called "prime rendering". We then learn the network parameters by backpropagating the loss function and obtaining a delta image that captures the gradient of the sensor image. Next, we repeat the differentiable raytracing and back-propagate the delta image to learn the lens parameters.

Another strategy for saving memory in differentiable raytracing is to divide the rendered image into small patches and render these patches separately. The optical rays do not interact with each other and intersect the lens independently, so the total gradient is the sum of the gradients of each ray. This strategy allows us to perform differentiable ray tracing for high-resolution sensors.

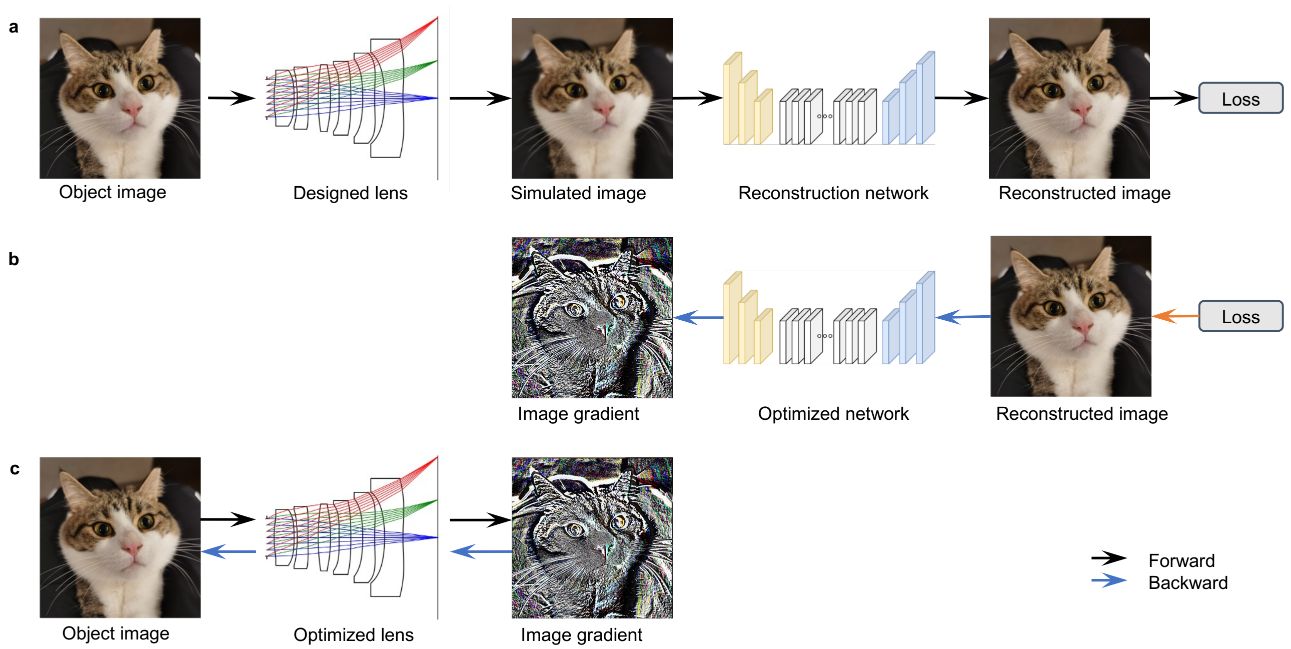

Supplementary Figure 3. Three stages in the adjoint rendering method. a Prime rendering. At this stage, we perform ray tracing without tracking gradient information. We simulate the sensor image and feed the image into the reconstruction network. **b** We back-propagate image errors to optimize network parameters and capture image gradient (delta image). **c** Adjoint rendering. We re-render differentiable raytracing with gradient tracking, then back-propagate the delta image to optimize the lens gradient.

Supplementary Note 2.2 Shape Correction

Due to the instability of the aspheric surface, abnormal geometries such as self-intersections often occur during training. Avoiding abnormal shapes is necessary for two main reasons: First, abnormal geometries cannot be

manufactured. Second, rays arriving at abnormal surface areas can back-propagate large gradients, leading to training crashes. To solve this problem, we used three methods. First, surface radius trimming after each epoch. Second, an obliquity regularization loss penalizes rays with large angles of incidence. Third, a thickness control loss to avoid self-intersection.

After each epoch, we trim the radius of each surface. The radius of the surface cannot be optimized during training, but a large radius usually causes abnormal shapes at the edge and affects the computation of Newton's method. Experimental results show that a reasonable radius makes the training process more stable by reducing the variance. To find a suitable radius for each surface, we first calculated the minimum radius that allows all valid rays to pass, and then slightly expanded the radius by 5%. This small expansion allows the rays to move towards the "invalid" regions in the next training.

The obliquity regularization loss can also help to avoid abnormal shapes. It works as follows: the obliquity regularization loss forces the surface to bend perpendicular to the incident rays so that the surface shape does not change excessively over the range of the ray distribution. Experimental results show that the obliquity regularization loss can help to achieve both a smooth surface shape and a smooth ray path, avoiding self-intersection.

Another thickness control loss is used to avoid self-intersection. Specifically, we focus on the self-intersection on the axis and on the edge. We maximize the thickness loss:

$$\mathcal{L}_{\text{thickness}} = \sum_{i=0}^{M-1} \min(z_{i+1}(r_{i+1}) - z_i(r_i), d_0), \tag{13}$$

where z is the surface height (sag) at the distance r, d_0 is the hard thickness constraint, and M is the number of lens surfaces. When $z_{i+1}(r_{i+1}) - z_i(r_i)$ is larger than d_0, this loss function will return all zero gradients. When $z_{i+1}(r_{i+1}) - z_i(r_i)$ is smaller than d_0, the thickness loss will maximum $z_{i+1}(r_{i+1}) - z_i(r_i)$ by adjusting the lens parameters. On the axis, $r_{i+1}, r_i = 0$, we do not allow two surfaces to overlap, so $d_0 = 0$. At the edge, r_{i+1}, r_i are determined by the previous radius trimming operation. We do not want the two surfaces to intersect, and we should also keep some space for manufacturing, so we can set d_0 to a small positive value.

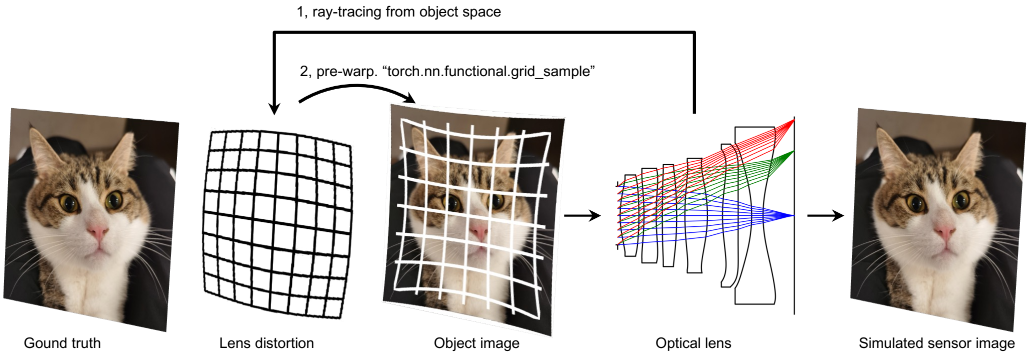

Supplementary Figure 4. Distortion relaxtion in differentiable ray traing. To get distortion-free sensor simulation, first we calculate the geometric distortion by ray tracing from the object space. For example, if the lens has a barrel distortion, we can get the barrel distortion map shown in the figure. Then we use the distortion map to resample the ground truth image, which is implemented with the PyTorch built-in `torch.nn.functional.grid_sample` function. The corresponding resampled image will present a pincushion distortion, which is the inverse of the barrel distortion. We use this "pre-warpped" image as the object image for image simulation. During the ray-tracing-based rendering process, two distortions will cancel out and the simulated sensor image will contain no distortion.

Supplementary Note 2.3 Distortion Relaxation

In order to relax distortion and enhance control over other optical aberrations during optimization, it is desirable to eliminate gradients resulting from distortion effects in back-propagation. Two approaches are available for this purpose: distorting the object image or undistorting the sensor image. In our experiments, we opted to pre-distort the object image to avoid the need for an intermediate undistortion operation in the end-to-end pipeline.

To apply distortion to the object image, we trace the chief ray for each sensor pixel and utilize the final position on the object plane as the reference for each sensor pixel. A straightforward approach is to warp the object image to match these reference points; however, this forward warping technique is challenging to implement. Instead, we propose an

alternative method of resampling the inverse mapping table. Specifically, we sample rays from the object plane, track them to the sensor plane, and compute the centroids of the rays to resample the object image. The resampling operation is notably simpler to implement using the PyTorch function `torch.nn.functional.grid_sample`. It is important to note that if the lens exhibits barrel distortion, the size of the pre-distorted object image should be increased.

Supplementary Note 3 Implementation Details of Automated Lens Design

Supplementary Note 3.1 Ray Sampling

When sampling rays from the image sensor, it is imperative to sample both their origin and direction.

For the origin sampling, we regard sensor pixels as points rather than squares, with rays originating from these points. During training, these rays are optimized, converging to the corresponding points on the object image. Consequently, the pixel-level image error matches the geometrical RMS error, ensuring that the gradient is accurately interpreted. Notably, since the sensor image and the object image are symmetrical relative to the lens's optical center, aligning the simulated image with the ground truth image on a pixel-by-pixel basis requires a specific approach. If we take the top-left point as the reference $I(0,0)$ in bilinear interpolation, then we should represent the sensor pixel using the bottom right point during ray sampling. Failing to do this will result in a one-pixel misalignment between the simulated sensor image and the object image, compromising optical performance metrics like the PSNR value.

Regarding direction sampling, we aim to enhance sampling efficiency and boost convergence speed. To achieve this, we first determine the exit pupil and subsequently sample points on it. The exit pupil, a virtual aperture in an optical system, is derived via ray tracing in our experiments. We then connect the sensor pixels to the sampled pupil points, normalizing the vectors to determine ray directions. If the number of samples per pixel (denoted as *spp*) is insufficient for uniform sampling across the pupil plane, we partition the exit pupil into regions of equal area and execute random sampling within these regions. For instance, when sampling 32 rays per pixel, we segment the exit pupil into 8 regions, drawing 4 points from each.

For each epoch, we maintain consistency by using the same set of rays. However, in the subsequent epoch, rays are resampled. This approach not only minimizes the overall sampling duration but also introduces variance during training. It's also worth noting that during adjoint rendering, there is no need to reset the pseudo-seed, as referenced in[12]. Instead of regenerating the same set of rays, they can simply be stored and reused.

Supplementary Note 3.2 Object Image and Distortion

The physical size of the object image should be scaled so that the simulated image can cover the entire sensor area, as shown in Fig. S1. The scale factor is calculated from the optical parameters and is fixed during training. In the beginning, we have no idea of a good design, so we assume that both the first and second principal points[13] is at the origin (the center of the first lens surface). Then the scale factor is α:

$$\alpha = \frac{d\tan(\theta)}{r}, \tag{14}$$

where d is the depth of the object image, r is half the diagonal length of the sensor or the image height, and θ is half the FoV. If the focal length is given instead of the image height, we can use FoV to convert the focal length to the image height.

At the beginning of training, we employ a static scale factor (perspective) and prohibit distortion. As a result, each sensor pixel retains a consistent reference in the object plane. Rays emanating from these sensor pixels are honed to converge upon these reference points. This robust "hard" reference addresses the challenge of traditional methods not converging during the initial stages of training.

During the fine-tuning phase, and with a solid design already in place, we can leverage ray tracing to determine a more precise scale factor. To do this, we sample rays from the sensor, trace them through to the object plane and ascertain the scale factor by computing the ratio between the sensor coordinates and the object plane coordinates. To mitigate distortion's influence, our sampling focuses primarily on the sensor's central region. Experimental findings indicate an approximate difference of $\sim 2\%$ between the scale factors derived from both methodologies. Additionally, in the fine-tuning phase, we allow for some degree of distortion, affording us enhanced control over other optical aberrations.

To facilitate interpolation for edge pixels, we pad the object image by one pixel. We have observed that this padding can markedly elevate the optical performance of edges in the final outcome. Absent this padding, the rays at the edges remain suboptimal. It's worth noting that certain images feature expansive solid-color regions, often in the background. These homogeneous expanses fail to yield sufficient gradients, leading the training to become entrapped in local minima. To counteract this issue, we introduce minor wavelet noise into the training images.

Supplementary Note 3.3 Dataset

We employ the DIV2K dataset[14] for our experiments. Nonetheless, any image can serve as training data, as long as it encompasses a sufficient mix of high and low-frequency information. We remain indifferent to the semantic

content. While we have experimented with random sinusoidal and Gaussian noise for training purposes, we found them less effective compared to authentic images. The DIV2K dataset comprises 800 training images and 200 test images. For each training epoch, we crop and resize these images to align with the sensor's resolution. Given that some images have extensive solid color backgrounds, we introduce a minimal amount of wavelet noise. We simulate the RGB channels individually using distinct ray wavelengths, later integrating them to produce the final simulated image. For optimization, we chose the AdamW optimizer[15], and for adjusting the learning rate, we utilized the CosAnnealing learning rate scheduler[15]. Each training phase is initialized with a unique learning rate. Throughout the training, we typically maintain a batch size of 8.

Supplementary Note 3.4 Automated Lens Design Implementation

Beginning with several planes, we optimize an imaging lens autonomously. The optimization variables encompass curvature (c), the conic term (k), polynomial terms (α_i), and position (d). However, the number of lens elements and the material designated for each lens element are predefined and remain constant. We select from frequently used plastic materials suitable for smartphone lenses, including PMMA, COC, POLYSTYR, POLYCARB, OKP4, and OKP4HT. Another strategy for material selection could involve using proven combinations from existing patents. As our optimization hinges on the sensor image, we employ design targets like image height in lieu of focal length, along with FoV and aperture size. To mitigate chromatic aberration, we segment the full spectrum into three wavelengths (486 nm, 587 nm, and 656 nm) and simulate corresponding image channels.

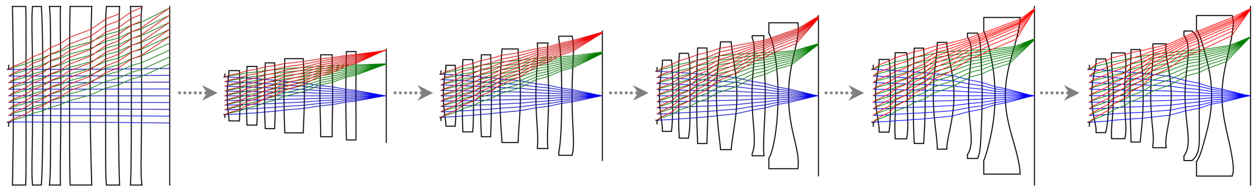

Supplementary Figure 5. An automated lens design example with curriculum learning. Starting from several planes, we first design a relatively simple lens (47.5°, F/2.8), then gradually increase FoV and aperture size to the final target (80.8°, F/2.0).

For example, consider the design of a 6-element lens with a FoV of 80.8°, an image height of 7.66 mm, and F/2.0. For a lens composed of 6 elements and polynomial terms ranging from α_2 to α_{12}, there are 108 optimizable variables (9 per surface for 12 surfaces). As illustrated in Fig. S5, we leverage the curriculum learning approach to design the lens in stages. Initially, our design objective is a FoV of 47.5° and F/2.8. Given the moderate design complexity at this stage, we can achieve a commendable design simply through weight masking and angle regularization. During this phase, we disregard sensor noise and vignetting to maintain accurate gradients. After each epoch, lens shape corrections are made to address invalid surfaces and self-intersections. Here, the adopted parameters are an image resolution of 256×256, an spp of 32, and a batch size of 16. Subsequently, we elevate the target FoV, F-number, and sensor resolution while retaining the same sensor size. Concurrently, lens thickness is reduced to ensure a seamless optical path. Given that the design challenges incrementally rise at each step, a satisfactory design is always attainable. Ultimately, we escalate the design parameters to the initial goals of FoV 80.8° and F/2.0. These stages constitute the main training. During main training, various learning rates such as $lr_c = 1e^{-4}$, $lr_k = 1e^{-1}$, $lr_d = 1e^{-4}$, and $lr_{\alpha_2} = 1e^{-4}$ are employed, with an exponential decay of 0.1 for higher polynomial terms ($lr_{\alpha_4} = 1e^{-5}$, $lr_{\alpha_6} = 1e^{-6}$, and so forth). The angle regularization term incorporates a weight factor of 4, and each stage is trained for 100 epochs. It's pertinent to highlight that the design path in the curriculum can be fairly flexible, the only constraint being that the incremental difficulty should be manageable.

Post establishing the FoV and aperture size to the design objective, a fine-tuning phase is initiated to further elevate imaging performance. During this phase, distortion constraints are relaxed and angle regularization is omitted. All learning rates are slashed by a factor of 0.1, and the results are fine-tuned at a high resolution (2048×2048) over 50 epochs.

Supplementary Note 3.5 PSF Calculation

After lens design, we evaluate the optical performance with the point spread function (PSF) which characterizes the optical lens response to a point source of light. Ray tracing through optical lenses is a well-established technique for obtaining a more accurate point spread function (PSF)[2,16–18]. This involves tracing a group of rays from a

point source through the lens group to the sensor plane, resulting in a spot diagram. We can then convert the spot diagram into sensor pixels and obtain the PSF by:

$$\mathrm{PSF}(\mathbf{o_p}) = \sum_{k=1}^{spp} u_k \cdot \sigma(|(\mathbf{o_p} - \mathbf{o_k}) \cdot \hat{\mathbf{e}}_x|/L) \cdot \sigma(|(\mathbf{o_p} - \mathbf{o_k}) \cdot \hat{\mathbf{e}}_y|/L), \tag{15}$$

where $\mathbf{o}_p$ denotes the coordinate of the pixel, and $\mathbf{o}_k$ represents the intersection point of the kth ray with the sensor plane. The variable *spp* stands for "samples per pixel", corresponding to the number of rays emitted from each point source, which is set to 2048 in our experiments. We assume that the energy of each ray, denoted by u_k, is equal to 1. $\hat{\mathbf{e}}_x$ and $\hat{\mathbf{e}}_y$ are unit vectors in the sensor plane, and L denotes the physical width of a sensor pixel. The σ function is defined as:

$$\sigma(x) = \begin{cases} 1 - x & 0 \le x \le 1 \\ 0 & \text{otherwise} \end{cases}, \tag{16}$$

which assesses a ray's impact on its surrounding pixels, with a greater impact attributed to rays in closer proximity. The total impact of a ray on the four surrounding pixels sums to one. By leveraging sub-pixel information, the σ function can more accurately represent the actual light distribution using a limited number of samples. A visualization of PSF calculation is presented in Fig. S6.

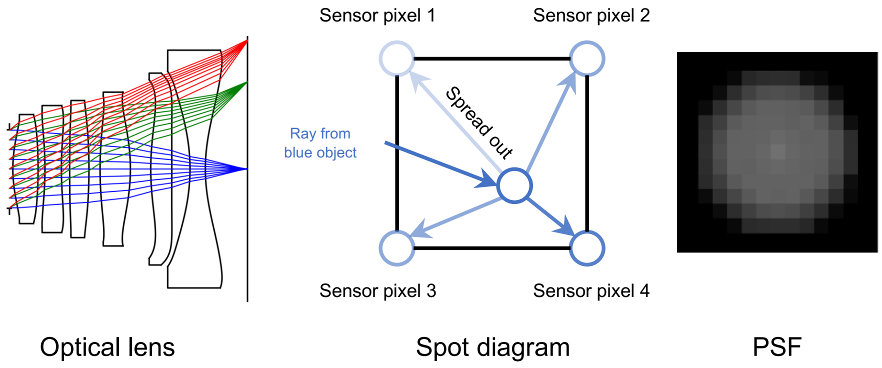

Supplementary Figure 6. PSF calculation with spot diagram. The intersection points of the rach light ray are divided into pixel bins to calculate the PSF. In our experiments, we does not use the PSF for image simulation, instead, ray tracing from sensor pixels is used to simulate the sensor image. The PSF is calculated for evaluation purposes only.

Supplementary Note 4 Additional results for automated lens design

Supplementary Note 4.1 Optical Performance Evaluation for Lens Design Example in Main Paper Fig. 1d

In Fig. S7, we present the optical performance evaluation for each intermediate step in the automated lens design process (Fig. 1d in the main paper). Throughout the optimization, the RMS spot size consistently decreases until it reaches a minimal value in the final design. The intermediate designs do not necessarily exhibit optimal optical performance, as the curriculum learning strategy aims to guide the automated lens design process toward finding a successful lens design pathway. In the last step, we relax the geometric distortion and fine-tune the lens to achieve the best optical performance. For the final design, it reaches an RMS spot error of less than 11 μm across various incident fields. This demonstrates promising optical performance, especially considering that our optical lens was designed automatically from scratch without any human intervention.

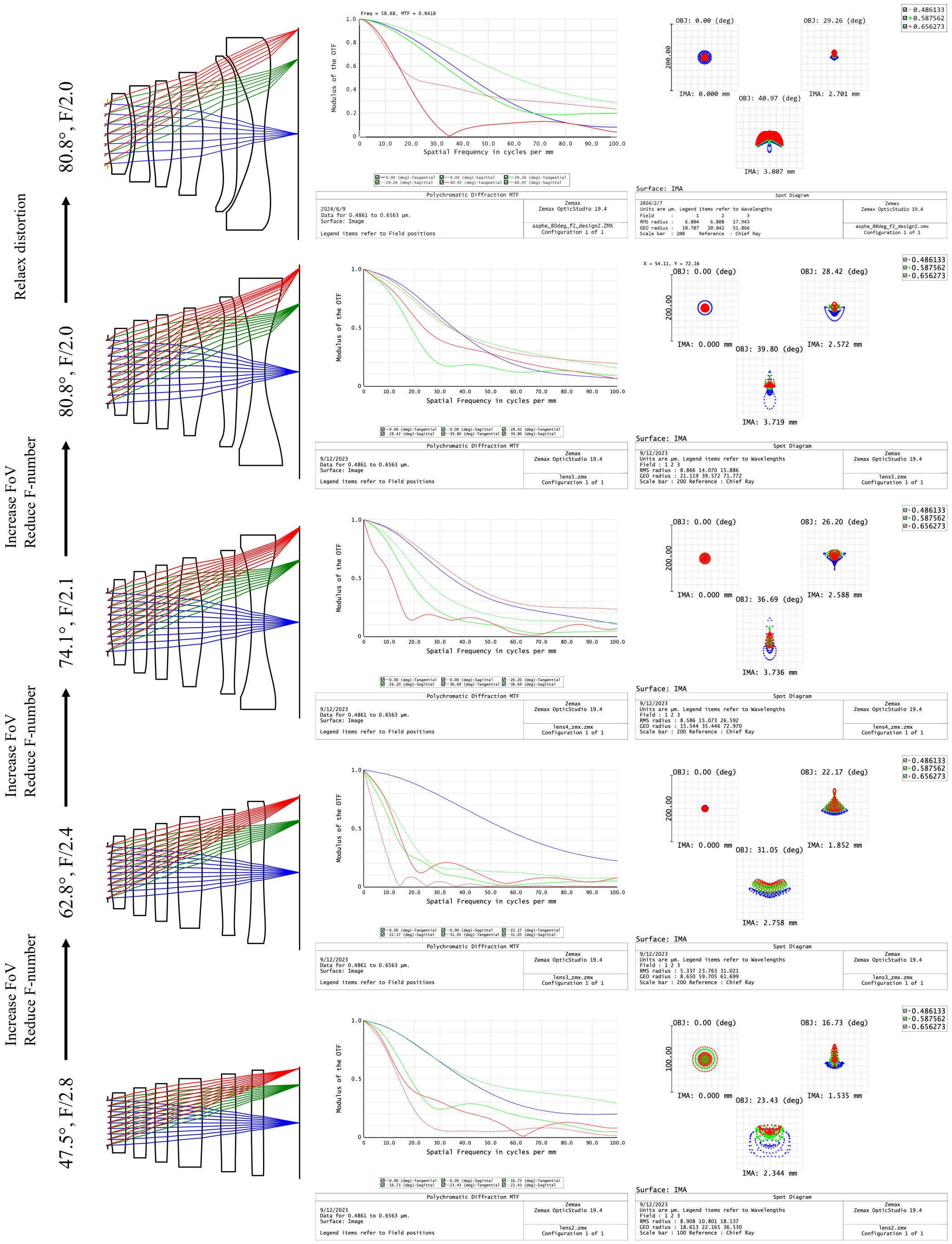

Supplementary Figure 7. Optical performance evaluation at each intermediate step. The RMS spot size of the lens decreases throughout the optimization, reaching a promising small value in the final design.

Supplementary Note 4.2 Automated Lens Design Results for Main Paper Table 1

In Table 1 in the main paper, we conduct an experiment to evaluate the effectiveness of different settings in the curriculum learning strategy. We select 20 random initial structures for automated lens design. The final designs from various experiments are presented in Fig. S8, S9, S10, and S11.

The basic differentiable ray tracing method, as presented in dO[2], fails to produce successful lens designs without self-intersection. This failure can be attributed to the need for careful control of highly aspheric lens surfaces. Introducing an optical regularization loss can help mitigate self-intersection issues, but the final designs may still suffer from local minima, particularly when large FoV optical rays fail to converge effectively.

The lens design curriculum, however, proves beneficial in guiding the automated lens design process to learn how to create high-performance lenses from scratch. Nevertheless, challenges persist, such as the occurrence of degenerated structures like self-intersection. By combining the lens design curriculum with the optical regularization loss, the automated lens design process can acquire the capability to design high-performance lenses from scratch.

Furthermore, when integrating the lens design curriculum, optical regularization loss, and re-weighting mask, the automated lens design process demonstrates even better results in designing high-performance lenses from scratch. This combination results in improved convergence of optical rays in the final designs, leading to lower Avg RMS spot sizes.

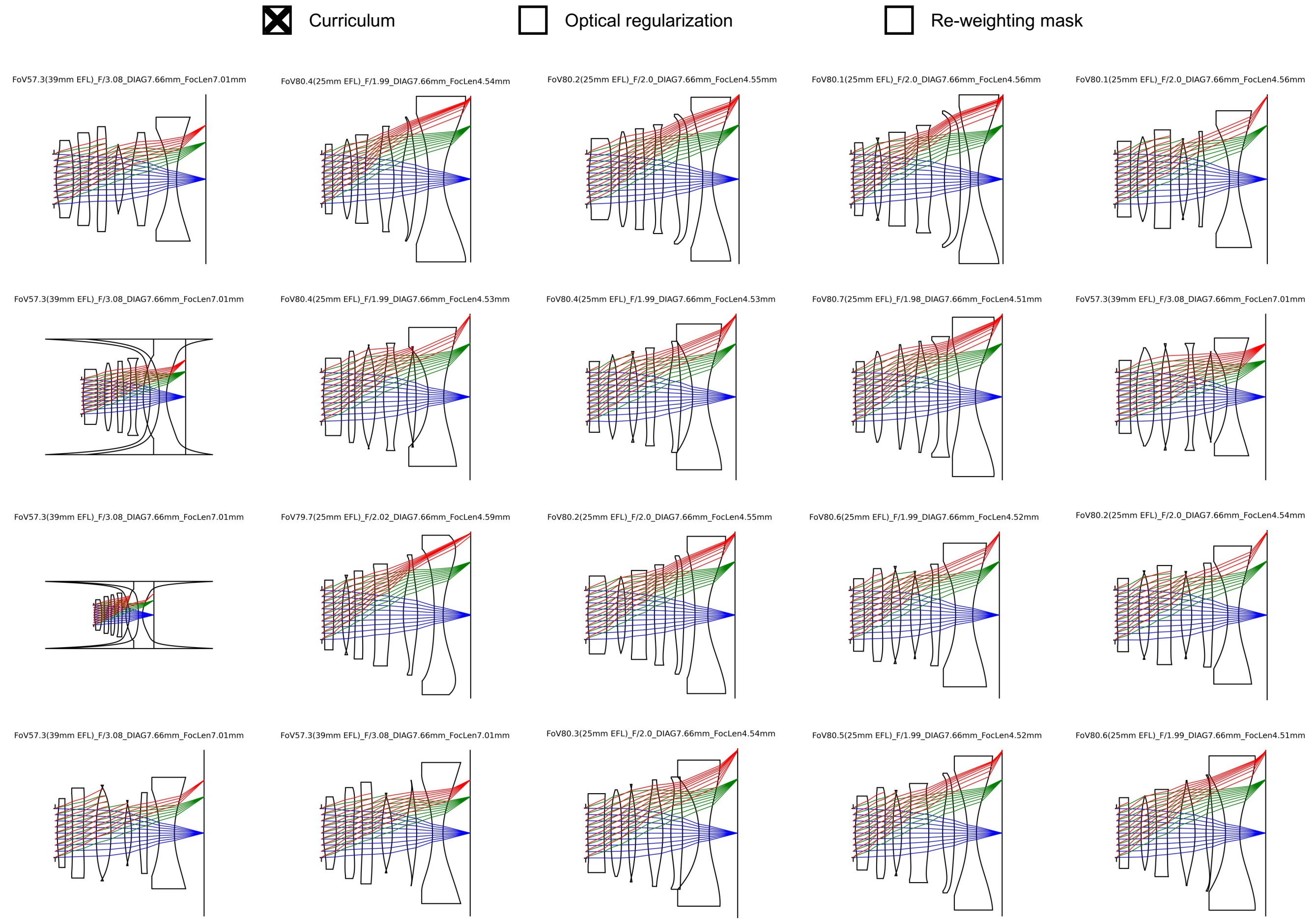

Supplementary Figure 8. Automated lens design results using the lens design curriculum, but without optical regularization or re-weighting mask. Through the progressive increase in lens design task difficulty, the curriculum learning approach can master the design of high-performance lenses from scratch. However, the lens design process may encounter issues, such as degenerated structures like self-intersection.

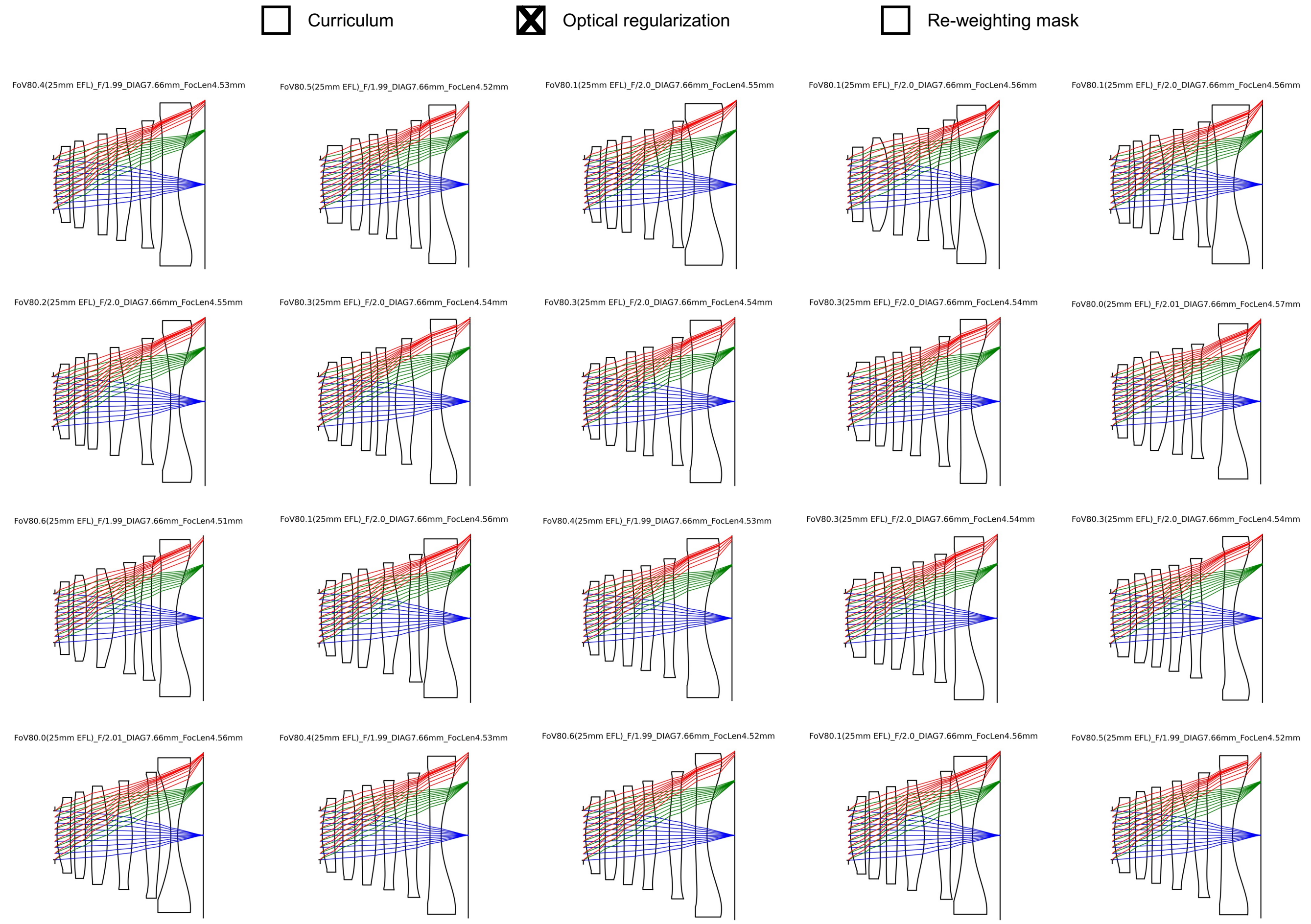

Supplementary Figure 9. Automated lens design results using optical regularization, but without lens design curriculum or re-weighting mask. By incorporating the optical regularization loss, the design process can prevent self-intersection. However, the final designs may encounter local minima issues, where large FoV optical rays do not converge effectively.

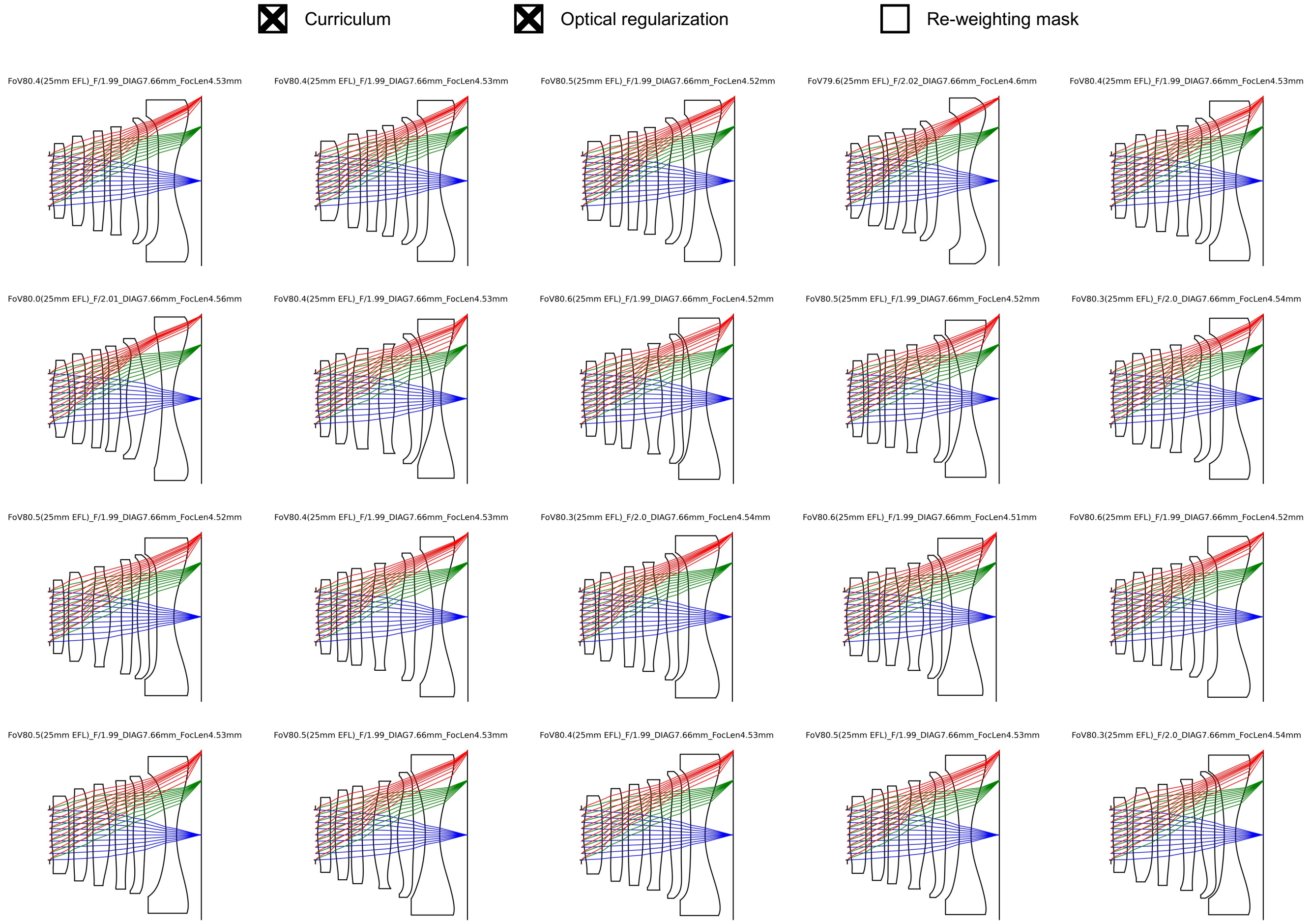

Supplementary Figure 10. Automated lens design results incorporating both lens design curriculum and optical regularization, but without a re-weighting mask. By integrating the lens design curriculum with the optical regularization loss, the automated process can efficiently design high-performance lenses from scratch.

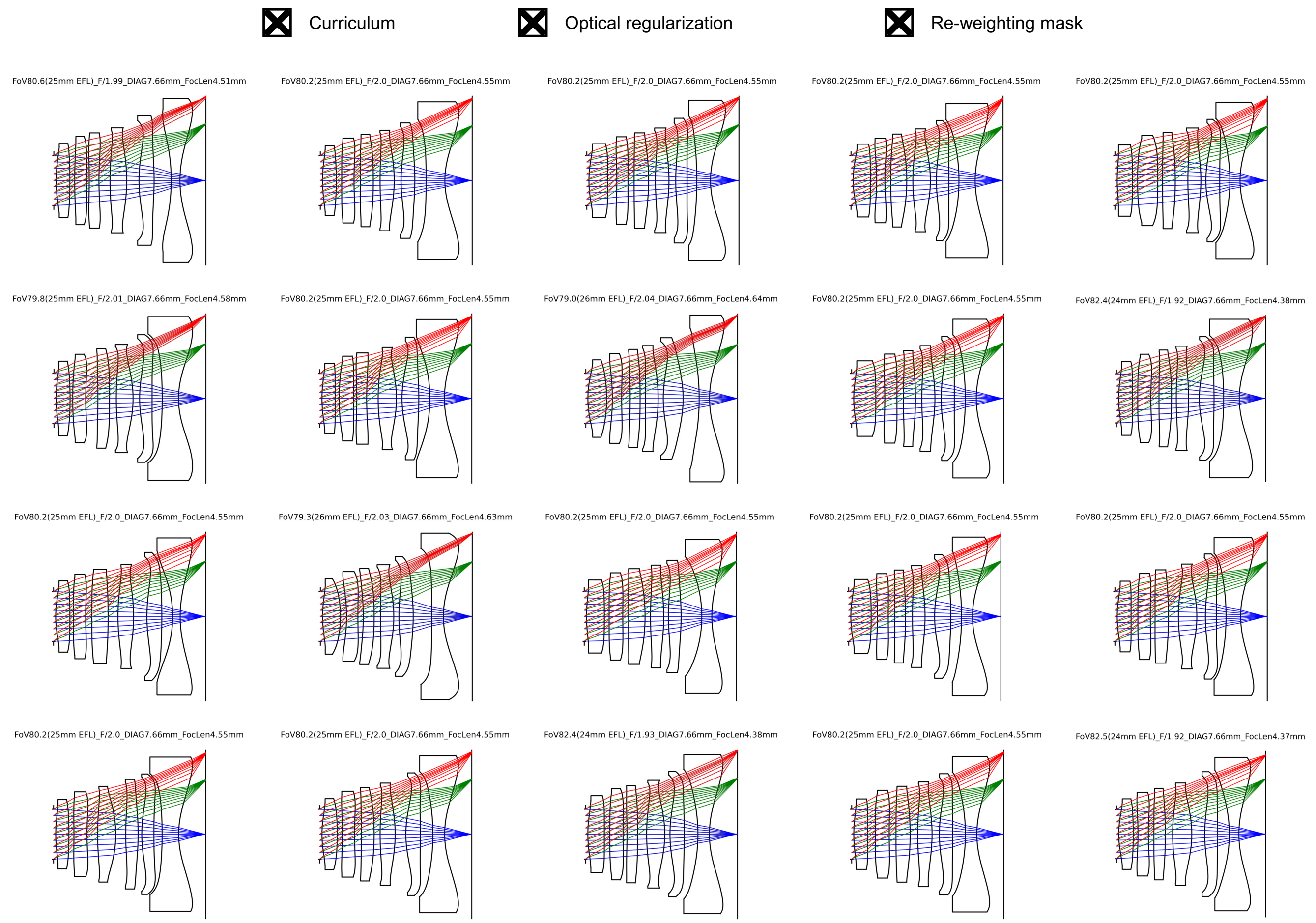

Supplementary Figure 11. Automated lens design results incorporating lens design curriculum, optical regularization, and re-weighting mask. By integrating these three elements, the automated lens design process is enabled to design high-performance lenses from scratch. This results in better convergence of the optical rays in the final designs, leading to a reduced Avg RMS spot size.

Supplementary Note 4.3 A Lens Design Example for 16 Mega-pixel Cellphone Camera

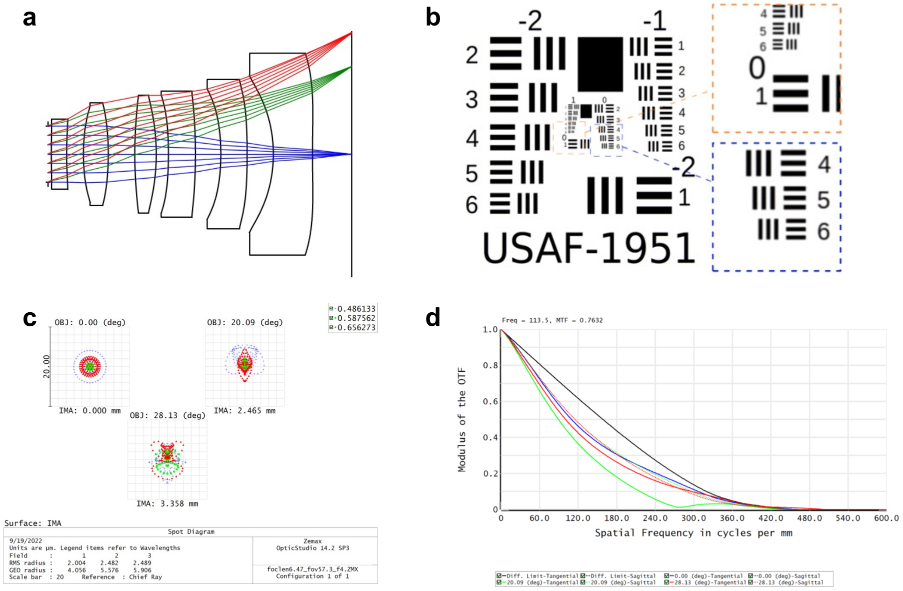

Supplementary Figure 12. Optical analysis of a lens designed by our proposed method. a Lens model with light path. **b** Simulated raw captured image at 10 m. The distortion of the image has been corrected in post-processing. **c** MTF curve of the lens (raw capture) at three fields. **d** Spot diagram of three wavelengths at three fields. **a**,**c** and **d** are generated in ZEMAX, and **b** is simulated raw capture by ray-tracing based rendering.

Supplementary Table 1. Lens data for Fig. S12a.

Surface	Radius	Thickness	Material	Semi-diameter	Conic	α_2	α_4	α_6	α_8	α_{10}	α_{12}
1	-93.39	0.433	OKP4	0.800	0.670	0.036	-0.025	-2.876e-3	1.813e-4	3.413e-4	2.614e-4
2	26.52	0.469		1.005	10.410	0.030	-0.014	-1.216e-3	1.000e-4	1.338e-4	-1.586e-4
3	44.08	0.742	PMMA	1.385	3.850	0.072	0.011	-4.667e-4	-1.098e-4	-5.147e-5	-3.879e-6
4	-57.87	0.779		1.465	3.110	-0.052	-6.448e-3	-1.720e-4	-1.775e-5	2.290e-5	4.234e-6
5	33.161	0.618	COC	1.680	5.390	0.027	-2.181e-3	-2.211e-4	9.789e-6	1.997e-5	1.051e-5
6	-26.34	0.344		1.705	2.880	-0.064	-7.112e-4	1.139e-4	1.056e-4	3.191e-5	4.570e-6
7	564.58	0.799	POLYCARB	1.705	2.880	-0.044	-9.870e-3	-5.598e-4	-1.048e-6	1.395e-5	6.905e-6
8	266.52	0.726		1.845	2.880	-0.031	-1.119e-3	-4.588e-4	1.341e-5	2.773e-5	9.080e-6
9	-165.24	0.812	POLYCARB	1.925	0.140	-0.077	-8.639e-3	-5.004e-4	1.284e-6	1.225e-5	2.554e-6
10	-122.50	0.750		2.335	0.170	-0.023	-5.448e-3	-5.920e-4	-3.270e-5	6.387e-6	5.237e-6
11	-20.66	1.168	OKP4	2.440	3.040	-0.103	-2.630e-3	-2.310e-4	1.100e-6	6.402e-6	2.819e-6
12	27.78	1.109		2.985	3.040	-3.607e-3	-5.133e-3	-1.604e-4	2.693e-5	2.499e-6	-3.392e-7
Sensor				3.500							

Specifically, we present a 6-element smartphone lens designed using our curriculum learning approach, as illustrated in Fig. S12. At the optimization's outset, all lens surfaces are initialized to be nearly flat and are randomly positioned. For each lens surface, we optimize curvature, position, conic, and polynomial terms from α_2 to α_{12}. During differentiable ray tracing, sensor noise and the vignetting effect are disregarded as they might distort the correct optical gradients for lens design tasks. The entire process takes about 24 h (main training: $\sim$8 h; fine-tuning: $\sim$ 12 h) on a single A100 GPU.

As seen in Fig. S12a, the designed lens bears a resemblance to the shapes of commonly used commercial smartphone lenses, even though no prior knowledge or human intervention was incorporated during the entire training process. The design targets are a focal length of 7.00 mm, FoV 57.3°, and F/4. The lens boasts a total thickness of 8.68 mm, an effective focal length of 6.47 mm, and a back focal length of 2.73 mm. Furthermore, the lens has a FoV of 56.8°, an image height of 7.00 mm, and a numerical aperture of F/4. To assess the optical performance of our designed lens, we conducted analyses using simulated images and optical objectives. As depicted in Fig. S12c

and d, the MTF value of the central field at 100 lp/mm stands at 54%. Across the full FoV zone, the MTF value at 100 lp/mm exceeds 50% in the sagittal plane and 40% in the tangential plane. At 200 lp/mm, the MTF value surpasses 20% in the sagittal plane and 10% in the tangential plane. The RMS spot radius for three fields (0, 0.707FoV, and FoV) measures 2.004 μm, 2.482 μm, and 2.489 μm, respectively, which is sufficient for 16-megapixel imaging. This indicates that the entire FoV can be imaged with exceptional clarity. Fig. S12b showcases the simulated sensor image of the USAF-1951 resolution test target from a distance of 10 m, with magnified details. Post-processing corrected the distortion in the simulated image. Notably, the lens can discern the finest lines on the resolution chart, which have a physical separation of approximately 5 cm at a 10 m distance. Table. S1 lists detailed data for the lens. Four materials were randomly chosen for the lens's construction.

Supplementary Note 4.4 More Automated Lens Design Examples

Extensive experimental results demonstrate that our proposed curriculum learning strategy can design compound imaging lenses from scratch without any external intervention. We provide additional examples (Fig. S13 to S18) of lenses designed using our approach. The FoV, F-number, and image height are set as design targets.

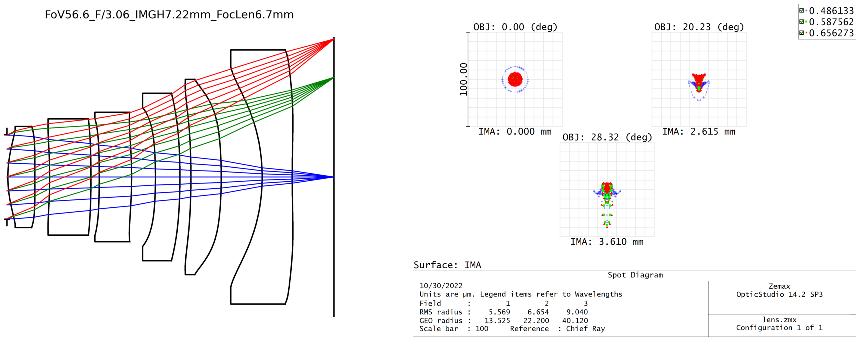

Supplementary Figure 13. Automated lens design example 1.

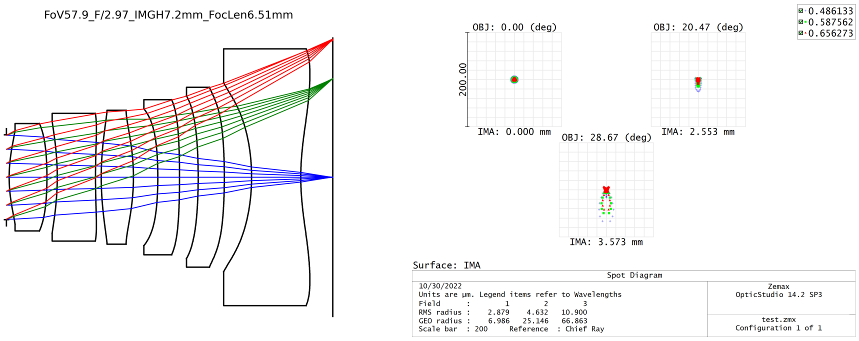

Supplementary Figure 14. Automated lens design example 2.

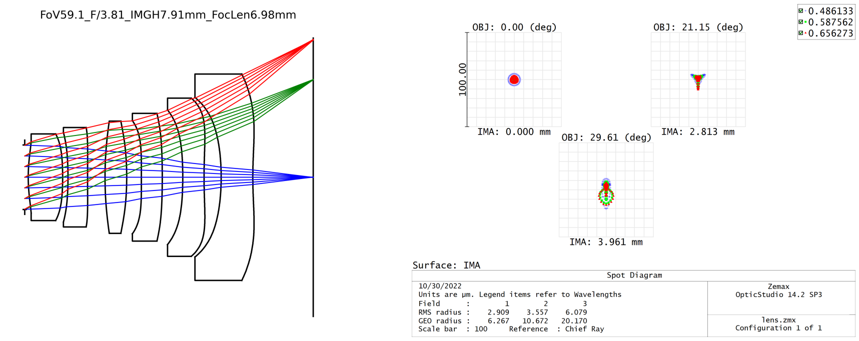

Supplementary Figure 15. Automated lens design example 3.

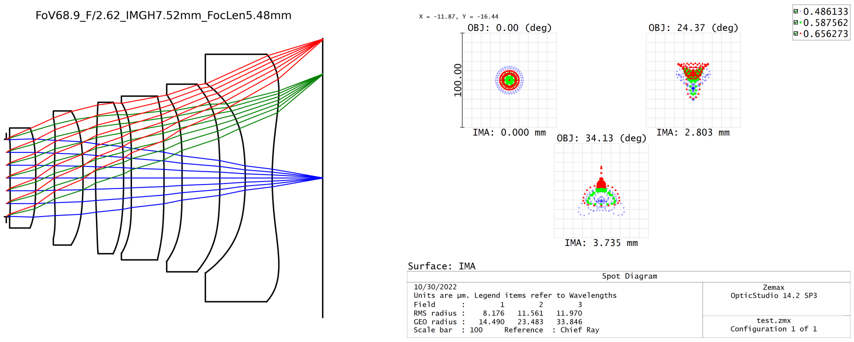

Supplementary Figure 16. Automated lens design example 4.

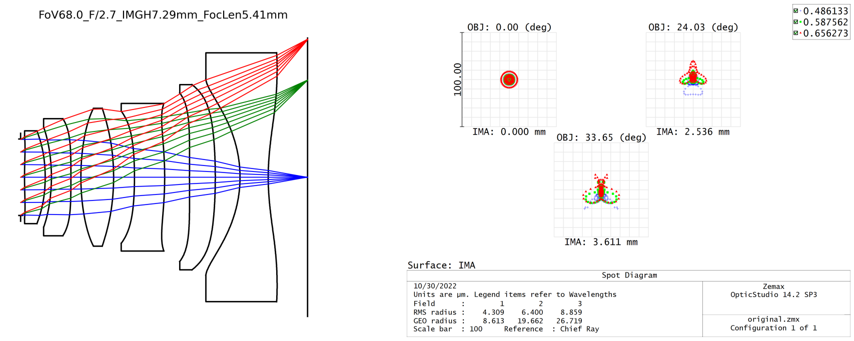

Supplementary Figure 17. Automated lens design example 5.

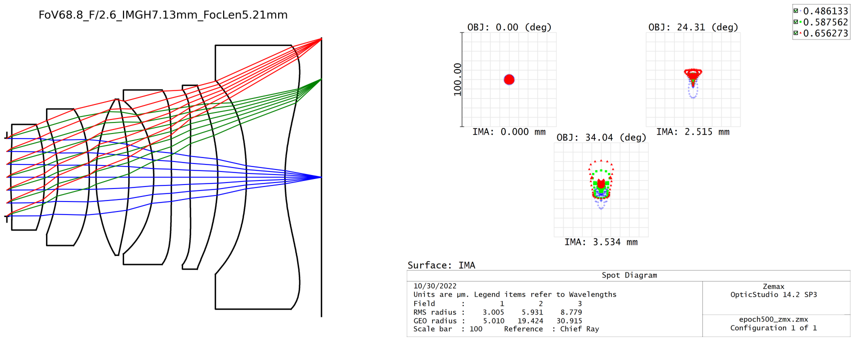

Supplementary Figure 18. Automated lens design example 6.

Supplementary Note 5 Additional Results for EDoF Lens Design

Supplementary Note 5.1 End-to-end Training for Computational Lens Design

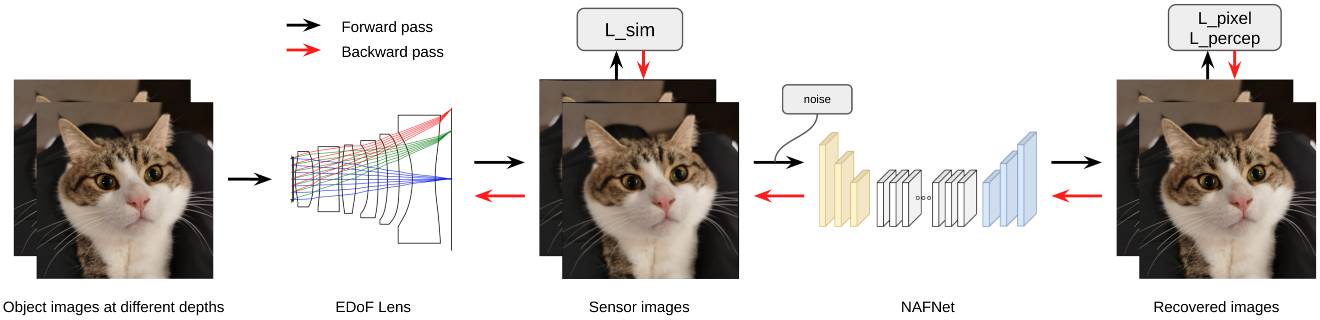

Supplementary Figure 19. End-to-end training pipeline. We jointly optimize the EDoF lens and the image reconstruction network to produce clear images within the extended depth range. A similarity loss is used to help get depth-independent imaging results. A pixel and a perceptual loss are used for the best output quality.

To design a computational lens from scratch, we first design an imaging lens and then design the computational lens based on it. Following the idea of curriculum learning, the difficulty increments at each step are not too large. For EDoF imaging, two options are adopted: the first is to use an odd polynomial surface in front of the lens, and the second is to replace an aspheric surface with a hybrid aspheric-odd polynomial surface:

$$z(r) = \underbrace{\frac{r^2}{R\left(1+\sqrt{1-(1+\kappa)r^2/R^2}\right)} + \alpha_2 r^2 + \alpha_4 r^4 + \cdots +}_{\text{aspheric}} \underbrace{\sum_{i=1}^{n}(a_i x^{2i+1} + b_i y^{2i+1})}_{\text{odd-polynomial}}, \tag{17}$$

where only a_i and b_i are optimizable lens parameters during the End2End training. It is worth noting that our differentiable ray tracing can apply to any type of refractive surface, so it can also handle this hybrid surface.

The implementation details and experiment settings mirror those used in automatic lens design. As illustrated in Fig. S19, we first simulate sensor images and then input these images into the image reconstruction network. During the end-to-end training stage, we overlook factors like sensor noise, vignetting effects, and the ISP process, as they typically are not relevant in the lens design phase. Valid lens design gradients are drawn from the optics and network components, not from sensor noise and other disturbances. These latter elements can skew optimization by providing biased and imprecise gradients. Our loss function for EDoF imaging is defined as:

$$\mathcal{L} = \sum_{d \neq d'} \mathcal{L}_{\text{sim}}(\widetilde{I}_d, \widetilde{I}_{d'}) + \sum_{d} \left[\omega_1 \mathcal{L}_{\text{raw}}(\widetilde{I}_d, I) + \omega_2 \mathcal{L}_{\text{recon}}(\bar{I}_d, I)\right], \tag{18}$$

where I, $\widetilde{I}$ and $\bar{I}$ represent the object image, simulated image, and reconstruction results, respectively. Hyperparameters ω_1 and ω_2 are used to balance different loss terms. In our experiments, we set ω_1 and ω_2 both to 0.1. At this stage, we optimize all optical and network parameters to learn similar image simulations across varying depths ($\mathcal{L}_{\text{sim}}$), while also achieving the best simulation quality ($\mathcal{L}_{\text{raw}}$) and the compatibility with the downstream deep network ($\mathcal{L}_{\text{recon}}$). In our experiments, we employ the SSIM loss as the similarity loss ($\mathcal{L}_{\text{sim}}$) and the simulation loss ($\mathcal{L}_{\text{raw}}$). For the reconstruction loss ($\mathcal{L}_{\text{recon}}$), we use the l_2 loss as the pixel loss and the SSIM loss as the perceptual loss[19], which can be written as:

$$\mathcal{L}_{rec}(\bar{I}_d, I) = \mathcal{L}_{\text{pixel}}(\bar{I}_d, I) + \beta \mathcal{L}_{\text{percep}}(\bar{I}_d, I), \tag{19}$$

where β is set to 0.01 in our experiments. To conserve memory, we randomly select two depths in each iteration to simulate images as captured by the sensor, as illustrated in Fig. S19. In our experiments, we set $\alpha = 0.01$ and $\omega = 0.2$. The initial learning rates are $1e^{-3}$ for a_1 and b_1, $1e^{-4}$ for a_2 and b_2, and so forth. The initial learning rate

Supplementary Table 2. Reconstruction results of hybrid-surface EDoF lens with manufacturing and assembling errors.

Lens	100 mm	150 mm	200 mm	300 mm	500 mm	1000 mm	3000 mm	5000 mm	10000 mm
Designed	32.6755/0.9302	33.7975/0.9451	34.2992/0.9501	34.4033/0.9502	34.0311/0.9450	33.4260/0.9363	32.8742/0.9277	32.6502/0.9250	32.5214/0.9230
Lens #1	31.0528/0.9136	33.4283/0.9415	34.1620/0.9488	34.3636/0.9502	34.1408/0.9468	33.6104/0.9395	33.1089/0.9321	32.956/0.9302	32.8651/0.9288
Lens #2	32.1467/0.9267	33.5937/0.9440	34.1018/0.9490	34.1354/0.9485	33.7225/0.9427	33.0772/0.9334	32.4779/0.9241	32.3189/0.9216	32.1836/0.9195
Lens #3	31.1665/0.9155	33.4640/0.9421	34.1638/0.9491	34.3428/0.9502	34.1158/0.9467	33.5688/0.9392	33.0844/0.9320	32.9689/0.9302	32.8792/0.9288
Lens #4	30.7496/0.9099	33.3371/0.9409	34.0920/0.9485	34.2843/0.9498	34.0514/0.9463	33.5043/0.9389	33.0102/0.9315	32.8932/0.9298	32.8002/0.9283
Lens #5	30.2268/0.9033	33.1305/0.9377	33.9421/0.9466	34.1381/0.9479	33.9063/0.9445	33.3598/0.9371	32.8657/0.9297	32.7487/0.9280	32.6557/0.9265
Avg (#1 $\sim$ #5)	31.0685/0.9138	33.3907/0.9412	34.0923/0.9484	34.2528/0.9493	34.0074/0.9454	33.4241/0.9376	32.9094/0.9299	32.9771/0.9282	32.8768/0.9264

Designed: designed lens without manufacturing and assembling errors.
Lens #1 $\sim$ #5: designed lens with random manufacturing and assembling errors.
Avg (#1 $\sim$ #5): average of lens #1 $\sim$ #5.

for the network stands at $1e^{-4}$, and we operate with a batch size of 2. We conduct end-to-end training of the lens and network for 50 epochs which usually reaches convergence.

After end-to-end lens design, we fix the optical lens and fine-tune the network for the best performance. During this network fine-tuning phase, we incorporate white Gaussian noise with a standard deviation σ of 0.001. We also account for manufacturing and assembly errors by introducing minor perturbations to the lens data. The perturbations are added to the lens parameters. This approach aims to train our network to be more resilient to such deviations, thereby enhancing the robustness of our proposed models. We fine-tune the network for 100 epochs with a learning rate of $1e^{-4}$ and a batch size of 8.

Supplementary Note 5.2 Manufacturing and Assembly Error Analysis

To simulate common manufacturing and assembly errors, we intentionally introduce perturbations to each optical surface of the designed lenses. During the network inference stage, we use these perturbed optical lenses for image simulation, and then measure the reconstruction scores on these images that include errors.

Specifically, for aspherical surfaces, we add perturbations to lens radius r, curvature c, conic term κ, and aspherical coefficients a_i. For cubic-phase plate and hybrid surface, we add perturbations to the odd-polynomial coefficients a_i and b_i. The tolerance for each lens parameter is 0.1% of the designed value. Also, for cubic-phase plate and hybrid-surface, we add a random rotation to the lens element to simulate the assembly error. The rotation angle is randomly sampled from $[-0.5°, 0.5°]$. To address the random rotation of lens elements, instead of rotating the lens element itself, we rotate the incident ray positions ($\mathbf{o}$) before calculating intersections and refractions. This step is equivalent to lens element rotation. After calculating the intersection and refraction, an inverse rotation of the ray positions is performed to transform the ray positions back.

Since we can not determine the exact manufacturing and assembly errors for each individual lens in practice, we use the network model trained for the designed lens to infer various perturbed lenses. The reconstruction results are shown in Table 2, Table 3, and Table 4. For each lens design, we adopt five randomly perturbed lenses. From the tables, it can be seen that the reconstruction scores of the perturbed lenses are close to that of the designed lens, indicating that our network model is robust to manufacturing and assembly errors. Despite the perturbations, the reconstruction scores of our end-to-end designed EDoF lenses still surpass those of the classical lens.

Moreover, a recent literature[20] has demonstrated that the network fine-tuning process can further improve the robustness of the network to manufacturing and assembly errors.

Supplementary Table 3. Reconstruction results of cubic-phate EDoF lens with manufacturing and assembling errors.

Lens	100 mm	150 mm	200 mm	300 mm	500 mm	1000 mm	3000 mm	5000 mm	10000 mm
Designed	32.0099/0.9261	33.3354/0.9439	33.8960/0.9494	33.6368/0.9466	33.1517/0.9409	32.7427/0.9353	32.4182/0.9294	32.2786/0.9272	32.0809/0.9246
Lens #1	31.5875/0.9215	32.9741/0.9410	33.6576/0.9477	33.5991/0.9464	33.1891/0.9414	32.7503/0.9356	32.3865/0.9297	32.3236/0.9282	32.2129/0.9264
Lens #2	30.4876/0.9072	32.3885/0.9356	33.3038/0.9454	32.9684/0.9423	32.4918/0.9363	32.1868/0.9315	31.7732/0.9252	31.7718/0.9247	31.7723/0.9241
Lens #3	31.4464/0.9197	33.0145/0.9412	33.8236/0.9488	33.7070/0.9474	33.2757/0.9423	32.8973/0.9372	32.5033/0.9313	32.4721/0.9303	32.4154/0.9291
Lens #4	32.0993/0.9279	33.4115/0.9447	33.7945/0.9487	33.4122/0.9447	32.8987/0.9384	32.4339/0.9319	32.0954/0.9253	31.7913/0.9212	31.4626/0.9169
Lens #5	31.9709/0.9263	33.282/0.9437	33.8197/0.9488	33.6507/0.9465	33.2122/0.9412	32.7502/0.9351	32.4411/0.9292	32.2497/0.9265	32.0089/0.9235
Avg (#1 ~ #5)	31.5183/0.9205	33.0141/0.9412	33.6798/0.9479	33.4675/0.9455	33.0135/0.9399	32.6037/0.9343	32.2399/0.9281	32.1217/0.9262	31.9744/0.9240

Designed: designed lens without manufacturing and assembling errors.
Lens #1 ~ #5: designed lens with random manufacturing and assembling errors.
Avg (#1 ~ #5): average of lens #1 ~ #5.

Supplementary Table 4. Reconstruction results of classical lens with manufacturing and assembling errors.

Lens	100 mm	150 mm	200 mm	300 mm	500 mm	1000 mm	3000 mm	5000 mm	10000 mm
Designed	31.4607/0.9226	34.2714/0.9580	34.1254/0.9531	32.8962/0.9367	32.1205/0.9242	31.1501/0.9075	30.4399/0.8932	30.2852/0.8898	30.1525/0.8868
Lens #1	30.4772/0.9078	33.6510/0.9533	34.4451/0.9559	33.2116/0.9400	32.3808/0.9277	31.3939/0.9117	30.6132/0.8973	30.4688/0.8942	30.3643/0.8919
Lens #2	29.0654/0.8819	32.6723/0.9447	34.4144/0.9562	33.2945/0.9413	32.4438/0.9289	31.5162/0.9142	30.7168/0.9000	30.5615/0.8970	30.4489/0.8947
Lens #3	30.0509/0.9051	33.3331/0.9513	34.2381/0.9543	33.0049/0.9386	32.1829/0.9262	31.2061/0.9103	30.4861/0.8961	30.3314/0.8928	30.2089/0.8899
Lens #4	29.3774/0.8892	32.5609/0.9444	34.0301/0.9537	33.0577/0.9394	32.3563/0.9283	31.4991/0.9139	30.7834/0.9007	30.6361/0.8978	30.5303/0.8955
Lens #5	30.9026/0.9140	34.2108/0.9573	34.6455/0.9569	33.2503/0.9401	32.4280/0.9278	31.4272/0.9118	30.6519/0.8977	30.5078/0.8946	30.4083/0.8924
Avg (#1 ~ #5)	29.9747/0.8996	33.2856/0.9502	34.3546/0.9540	33.1638/0.9399	32.3584/0.9278	31.4085/0.9124	30.6503/0.8984	30.5011/0.8953	30.3921/0.8931

Designed: designed lens without manufacturing and assembling errors.
Lens #1 ~ #5: designed lens with random manufacturing and assembling errors.
Avg (#1 ~ #5): average of lens #1 ~ #5.

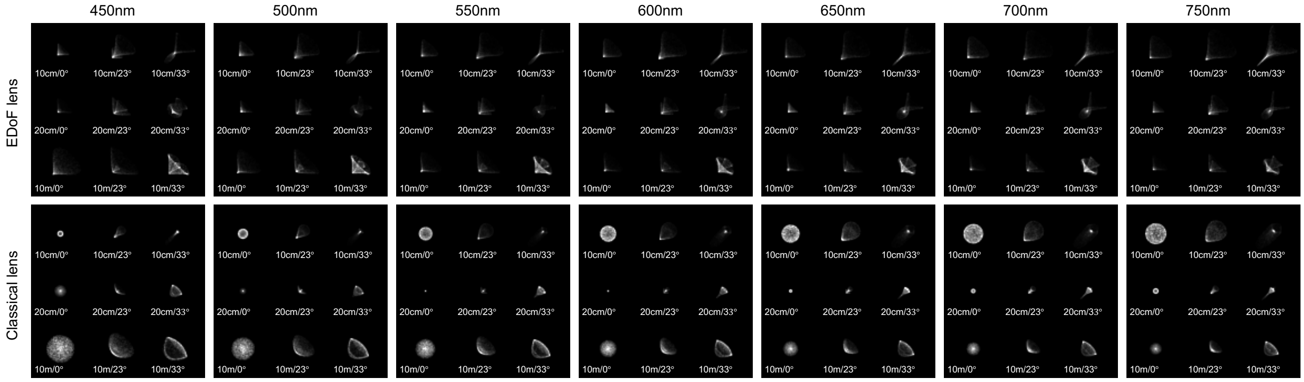

Supplementary Figure 20. Spectrum analysis of PSFs at different depths and view angles. Top: PSF at different depths and view angles of the EDoF lens presented in the main paper. Bottom: PSF at different depths and view angles of the classical lens presented in the main paper.

Supplementary Note 5.3 Spectrum Analysis

In our experiment, we employ three wavelengths to represent the full spectrum for EDoF training. This choice is motivated by the fact that commercial cameras typically use RGB image sensors, and the majority of image datasets utilized in deep learning contain only RGB images. A rigorous evaluation of optical performance across different wavelengths is provided in this section. The corresponding PSFs at varying depths and view angles are showcased in Fig. S20. Observing that the PSFs across different wavelengths are alike, suggests that the EDoF lens can generate consistent image simulations irrespective of the wavelength. Consequently, we contend that using three wavelengths as a representation of the full spectrum for EDoF training is justifiable. This approach substantially streamlines the experiment and curtails computational costs without markedly compromising simulation accuracy.

Supplementary Note 5.4 Comparison Between Hybrid and Cubic Plate EDoF Lenses

Conventional EDoF studies[21,22] utilizing cubic phase plates have demonstrated the capability to extend the depth of field. However, these studies typically idealize optical lenses as thin lenses without aberrations, necessitating a narrow FoV and small aperture size. This idealization does not hold in our context, where optical aberrations, particularly off-axis aberrations, are significant in lenses with a wide FoV and larger aperture size. These off-axis aberrations necessitate a more precise control over the cubic phase plate's height profile to achieve EDoF within a relatively large FoV. This is the reason for us to use of an odd-polynomial term of higher order than the conventional cubic phase plate.

In our experiments, both the hybrid lens and the cubic phase plate lens are trained using full-resolution images that cover the entire FoV. Detailed height profiles, along with corresponding lens figures and PSFs at various FoVs and depths, are presented in Fig. S21. The height profile of the odd polynomial term offers greater degrees of freedom, particularly for off-axis image regions, despite the differences are small. The PSF figures illustrate that the hybrid lens provides more uniform PSFs across different depths and FoVs.

To quantitatively evaluate the depth invariance across different FoVs, we measure the RMS error of the reconstruction results at different depths and image regions. We split the images into "center" and "edge" regions. In our experiments, the image resolution is 1024×1024, and the "center" region is defined as the central 512×512 pixels. The results are shown in Table S5. The hybrid lens significantly outperforms the cubic plate lens in terms of the RMS error at "edge" regions, which indicates that the hybrid lens is more robust to off-axis aberrations because the higher order of odd polynomial term provides more degree of freedom to control the height profile.

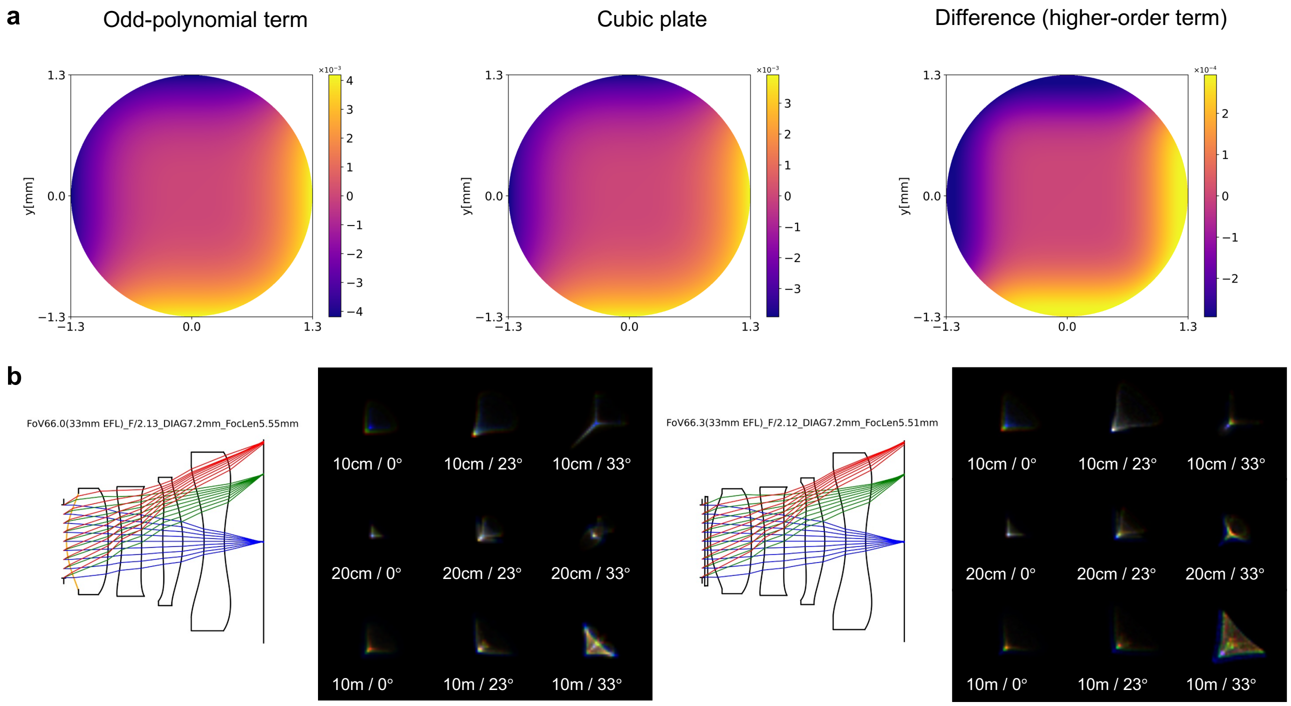

Supplementary Figure 21. Comparison between the hybrid and cubic plate EDoF lenses. a Height profiles of the hybrid and cubic plate EDoF lenses. **b** Lens figures and PSFs at different FoVs and depths of the hybrid and cubic plate EDoF lenses.

Supplementary Table 5. Comparison of RMS errors of "center" and "edge" regions between the hybrid and cubic plate EDoF lenses.

RMS error ($1e^{-3}$)	100 mm	150 mm	200 mm	300 mm	500 mm	1000 mm	3000 mm	5000 mm	10000 mm
Hybrid (center)	1.034	0.787	0.677	0.591	0.568	0.631	0.727	0.757	0.781
Cubic (center)	1.174	0.807	0.625	0.579	0.607	0.625	0.683	0.711	0.753
Hybrid (edge)	**0.631**	**0.506**	**0.462**	**0.481**	**0.557**	**0.654**	**0.742**	**0.776**	**0.798**
Cubic (edge)	0.728	0.574	0.541	0.610	0.691	0.760	0.825	0.854	0.891

Supplementary Note 5.5 More qualitative results

In Figs. S22, and S23, we present qualitative results comparing the classical lens, the EDoF lens with a cubic phase plate, and the EDoF lens with a mixed surface. Each of these lenses is paired with an image processing network trained to mitigate optical aberrations at varying depths. Among them, the EDoF lens with the mixed surface demonstrates the best output quality, indicating its ability to image clearly from 10 cm to 10 m. While the classical lens produces sharp images at the focal depth, but its image quality deteriorates at defocused depths compared to the EDoF lenses.

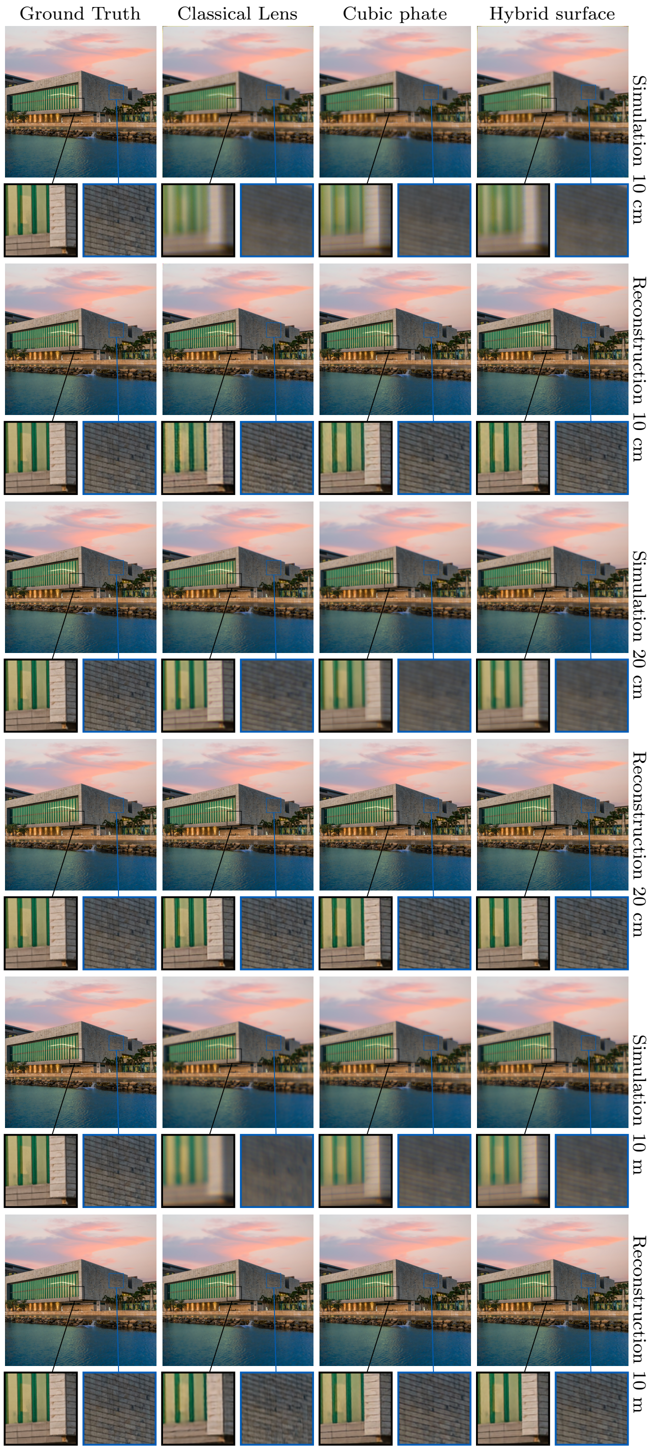

Supplementary Figure 22. EDoF results example 1. Photograph by Xinge Yang (CC BY 2.0).

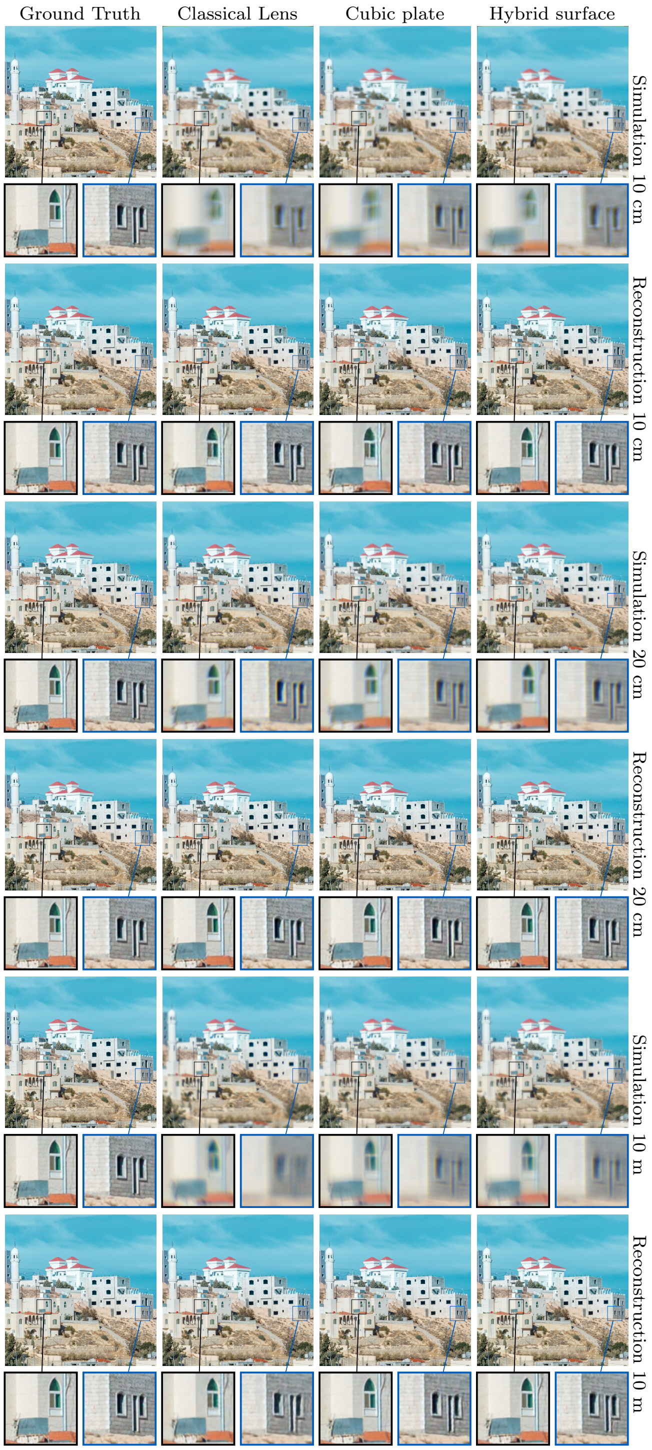

Supplementary Figure 23. EDoF results example 2. Photograph by Xinge Yang (CC BY 2.0).

References

1. Sun, Q., Wang, C., Qiang, F., Xiong, D. & Wolfgang, H. End-to-end complex lens design with differentiable ray tracing. *ACM Trans. Graph* **40**, 1–13 (2021).
2. Wang, C., Chen, N. & Heidrich, W. *dO*: A differentiable engine for Deep Lens design of computational imaging systems. *IEEE Trans. Comput. Imaging* (2022).
3. Sellmeier, W. Ueber die durch die Aetherschwingungen erregten mitschwingungen der Körpertheilchen und deren Rückwirkung auf die ersteren, besonders zur Erklärung der Dispersion und ihrer Anomalien. *Annalen der Physik* **223**, 525–554 (1872).
4. Tatian, B. Fitting refractive-index data with the Sellmeier dispersion formula. *Appl. Opt.* **23**, 4477–4485 (1984).
5. Jenkins, F. A. & White, H. E. Fundamentals of optics. *Indian J. Phys.* **25**, 265–266 (1957).
6. Fowles, G. R. *Introduction to modern optics* (Courier Corporation, 1989).
7. Peng, Y. *et al.* Learned large field-of-view imaging with thin-plate optics. *ACM Trans. Graph.* **38**, 219–1 (2019).
8. Sun, Q., Tseng, E., Fu, Q., Heidrich, W. & Heide, F. Learning rank-1 diffractive optics for single-shot high dynamic range imaging. In *Proceedings of the IEEE/CVF conference on computer vision and pattern recognition*, 1386–1396 (2020).
9. Tseng, E. *et al.* Differentiable compound optics and processing pipeline optimization for end-to-end camera design. *ACM Trans. Graph.* **40**, 1–19 (2021).
10. Nimier-David, M., Speierer, S., Ruiz, B. & Jakob, W. Radiative backpropagation: An adjoint method for lightning-fast differentiable rendering. *ACM Trans. Graph.* **39**, DOI: 10.1145/3386569.3392406 (2020).
11. Teh, A., O'Toole, M. & Gkioulekas, I. Adjoint nonlinear ray tracing. *ACM Trans. Graph.* **41**, 1–13 (2022).
12. Vicini, D., Speierer, S. & Jakob, W. Path replay backpropagation: Differentiating light paths using constant memory and linear time. *ACM Trans. Graph.* **40**, 108:1–108:14 (2021).
13. Smith, W. J. *Modern optical engineering: the design of optical systems* (McGraw-Hill Education, 2008).
14. Agustsson, E. & Timofte, R. NTIRE 2017 challenge on single image super-resolution: Dataset and study. In *The IEEE Conference on Computer Vision and Pattern Recognition (CVPR) Workshops* (2017).
15. Loshchilov, I. & Hutter, F. Decoupled weight decay regularization. *arXiv preprint arXiv:1711.05101* (2017).
16. Kolb, C., Mitchell, D. & Hanrahan, P. A realistic camera model for computer graphics. In *Proceedings of the 22nd annual conference on computer graphics and interactive techniques*, 317–324 (1995).
17. Côté, G., Mannan, F., Thibault, S., Lalonde, J.-F. & Heide, F. The differentiable lens: Compound lens search over glass surfaces and materials for object detection. In *Proceedings of the IEEE/CVF Conference on Computer Vision and Pattern Recognition*, 20803–20812 (2023).
18. LLC, Z. *Zemax User Manual* (2021).
19. Johnson, J., Alahi, A. & Fei-Fei, L. Perceptual losses for real-time style transfer and super-resolution. In *Computer Vision–ECCV 2016: 14th European Conference, Amsterdam, The Netherlands, October 11-14, 2016, Proceedings, Part II 14*, 694–711 (Springer, 2016).
20. Chen, S. *et al.* Computational optics for mobile terminals in mass production. *IEEE Transactions on Pattern Analysis Mach. Intell.* **45**, 4245–4259 (2022).
21. Dowski, E. R. & Cathey, W. T. Extended depth of field through wave-front coding. *Appl. Opt.* **34**, 1859–1866 (1995).
22. Sitzmann, V. *et al.* End-to-end optimization of optics and image processing for achromatic extended depth of field and super-resolution imaging. *ACM Trans. Graph.* **37**, DOI: 10.1145/3197517.3201333 (2018).
