# Supplementary material for: Curriculum learning for ab initio deep learned refractive optics
Source: Nat Commun. 2024 Aug 3;15:6572. doi: 10.1038/s41467-024-50835-7 (PMC11297943; doi:10.1038/s41467-024-50835-7)
Supplement: Supplementary file 3 — Description of Additional Supplementary Files [file 41467_2024_50835_MOESM3_ESM.pdf]

Description of Additional Supplementary Files

Supplementary Movie 1 - An automated lens design example.
